# Letter from O. B. Ormsby, Asst.-Surgeon, 18th Regt. Ill. Vols.

**Published:** 1862-05

**Authors:** O. B. Ormsby

**Affiliations:** Asst.-Surgeon; Pittsburg Landing, Tenn.


PITTSBURG LANDING, TENN., *April* 24, 1862.

DEAR DR.—I received a letter, a few days since, from my brother, in which he says that you say "write to me." I have. But I saw Prof. Andrews, one or two days since, and he said, "write to the Dr. giving him incidents of the battle," &c. I will begin by giving your a letter, the original of which is in my possession, word for word and letter for letter, except in the one item of names:—

"CAPTAIN ———, Ser, I have examined Mr Josuf ———, and find him unable to go into Survis at the presant time from debility of liver and disease which causes a ginerl debility of the hole sistem and I think he will not be able to go into Survis under 19 or 20 days. November the 23d 1861

Dr T. M. ————

In the presents of G. T. M———— J. P."

This I call decidedly rich; and it is not the only one of the kind I have seen either. They come to me too frequently. I presume from the "Ginerl debility of the *hole* sistem" mentioned in the letter the man must have diarrhœa; but the balance of the document borders on the mysterious.

I saw, during the recent battle, a very fine illustration of the difference in the effect of a cannon ball passing near the upper extremity or the lower. On Sunday the 6th inst. a man was brought in whose head had been very near the track of a cannon ball—which had not, however, disturbed even the hair. He was suffering very much from shock; perfectly insensible; the pupils dilated, &c.; in this condition he remained several hours. On the other hand, Capt. Anderson, of the 18th Ill. V. seeing a ball strike a horse, perhaps fifty yards in front of, and in a direct line from him, instantly leaped up from the ground, letting the ball pass under him, which it did, just touching his boot heels. He struck on his feet, however; and by stepping to one side, avoided the next one by an interval of four feet. Shock from a ball passing simply near one, without touching, is very strange. I have never seen it satisfactorily explained. I cut out a ball, which, entering the left breast, had carried in all the guard of a suspender buckle, and was found (guard and all) opposite the navel, on the right side. The man stood on his feet while I took the ball out; and he walked away very much pleased with my success in removing it. A ball struck the sleeve of a lieutenant's coat, which happened at the moment to be over the back of his hand. It was one of the large conical balls, but carried the coat before it, and burried itself in between the metacarpal bones, where it remained so firmly impacted that I was compelled to enlarge the opening before I could remove it. The coat was not torn but completely surrounded the ball.

A very great number of interesting and instructive incidents might be gathered; and I mean to gather up as many of them as I meet, which will, in my judgment, be of permanent value, and let you have those you wish for your Journal. In the meantime, I shall remember the more striking *incidents* that come under may notice; and if I live through the war, shall visit Chicago, when I shall probably have more to relate than you will care to listen to. Truly yours,

O. B. ORMSBY,
Asst.-Surgeon, 18th Regt. Ill. Vols.

